# The effect of targeted rheumatoid arthritis therapeutics on systemic inflammation and anemia: analysis of data from the CorEvitas RA registry

**DOI:** 10.1186/s13075-022-02955-y

**Published:** 2022-12-21

**Authors:** Anthony S. Padula, Dimitrios A. Pappas, Stefano Fiore, Taylor S. Blachley, Kerri Ford, Kelechi Emeanuru, Joel M. Kremer

**Affiliations:** 1 Northern California Arthritis Center, 120 La Casa Via, Suite 204, Walnut Creek, CA 94598 USA; 2 CorEvitas, Waltham, MA USA; 3 Sanofi, Bridgewater, NJ USA; 4 Evidera, Bethesda, MD USA

**Keywords:** Anti-rheumatic agents, Arthritis, Rheumatoid, Biological therapy, Inflammation

## Abstract

**Background:**

To evaluate the effects of tumor necrosis factor inhibitors (TNFi), interleukin-6 receptor inhibitors (IL-6Ri), and Janus kinase inhibitors (JAKi) on hemoglobin (Hb) and C-reactive protein (CRP) levels in adults enrolled in CorEvitas (formerly Corrona), a large US rheumatoid arthritis (RA) registry.

**Methods:**

Patients who initiated TNFi, IL-6Ri, or JAKi treatment during or after January 2010, had Hb and CRP measurements at baseline and 6-month follow-up (± 3 months) and had continued therapy at least until that follow-up, through March 2020, were included in the analysis. Changes in Hb and CRP were assessed at month 6. Abnormal Hb was defined as < 12 g/dL (women) or < 13 g/dL (men); abnormal CRP was ≥ 0.8 mg/dL. Differences in Hb and CRP levels were evaluated using multivariable regression.

**Results:**

Of 2772 patients (TNFi, 65%; IL-6Ri, 17%; JAKi, 17%) evaluated, 1044 (38%) had abnormal Hb or CRP at initiation; an additional 252 (9%) had both abnormal Hb and CRP. At month 6, the IL-6Ri group had a greater Hb increase than the TNFi (mean difference in effect on Hb: 0.28 g/dL; 95% CI 0.19–0.38) and JAKi (mean difference in effect on Hb: 0.47 g/dL; 95% CI 0.35–0.58) groups, regardless of baseline Hb status (both *p* < 0.001). The CRP decrease at month 6 was greater with IL-6Ri compared with TNFi and JAKi, regardless of baseline CRP status (both *p* < 0.05).

**Conclusion:**

These real-world results align with the mechanism of IL-6R inhibition and may inform treatment decisions for patients with RA.

**Supplementary Information:**

The online version contains supplementary material available at 10.1186/s13075-022-02955-y.

## Background

Rheumatoid arthritis (RA) is typically characterized by inflammation of synovial joint tissues, and symptoms may include extra-articular systemic manifestations including fatigue, anemia, weight loss, sleep disturbance, and depression [1, 2]. The pathophysiology of RA is associated with alterations in the cytokine network including increased serum and tissue levels of interleukin 6 (IL-6) [1, 3]. During the acute-phase response to tissue injury and inflammation in RA, there is a large increase in the synthesis of acute-phase reactants, including serum C-reactive protein (CRP), hepcidin, amyloid A, haptoglobin, ferritin, and plasma fibrinogen [4].

Anemia is a systemic manifestation of RA and is observed in approximately one-third of patients with RA. It correlates with an increased risk of physical disability and premature mortality [5, 6]. There is an association between anemia and fatigue [7], which is a key complaint in patients with RA [8]. IL-6 appears to be the central mediator of anemia in chronic disease, through its induction of hepcidin production [5, 9–11]. Hepcidin is a key regulator of systemic iron homeostasis, through its effects on iron resorption and subsequent erythropoiesis: high levels of hepcidin block intestinal iron absorption and iron recycling by macrophages, leading to iron sequestration, iron-restricted erythropoiesis, and anemia [1, 12]. IL-6 secretion results in decreased levels of transferrin, the primary iron transporter that delivers iron to the bone marrow for erythropoiesis [10]. Clinical studies have reported increased serum hepcidin in patients with RA, which was associated with elevated serum IL-6 and tumor necrosis factor alpha (TNF-α), suggesting a potential role of IL-6 inhibition in improving hemoglobin (Hb) levels in patients with RA [13–15].

High levels of serum IL-6 have also been associated with raised CRP levels, which is a clinical marker of inflammation [16] that is a significant predictor of radiographic damage in patients with RA [17, 18] and has also been linked to other systemic manifestations including cardiovascular disease (CVD) and depression [19].

Despite the known association of IL-6 with anemia and CRP levels in RA, only a limited number of small or post hoc studies have evaluated the impact of biologic or targeted synthetic disease-modifying anti-rheumatic drugs (bDMARDs or tsDMARDs) on Hb and CRP levels [15, 20–24]. To the best of our knowledge, no single study has compared the effects of the three drug classes, TNF-α inhibitors (TNFi), IL-6 receptor inhibitors (IL-6Ri), and Janus kinase inhibitors (JAKi), on Hb and CRP levels. We therefore undertook an assessment of the effects of TNFi, IL-6Ri, and JAKi on the levels of both Hb and CRP after 6 months of continuous therapy in adults enrolled in CorEvitas (formerly Corrona), a large US RA registry [25, 26].

## Methods

### Data source

The CorEvitas RA registry is an independent, prospective, national, observational cohort in which treatment and outcome data are collected from both rheumatologists and patients at the time of a clinical encounter (approximately every 6 months). Patients have been recruited from 208 private practices and academic sites across 42 US states, with 890 participating rheumatologists. As of September 2021, the CorEvitas RA registry included information on 57,300 patients. Data on 447,908 patient visits and 221,704 patient-years of follow-up observation time have been collected, with a mean patient follow-up of 4.7 (median 3.4) years. The characteristics of the CorEvitas RA registry have been described previously [25, 26].

All patients provided written informed consent and authorization before enrollment in the CorEvitas RA registry. This study was carried out in accordance with the Declaration of Helsinki. All participating investigators were required to obtain full institutional review board (IRB) approval for conducting non-interventional research involving human subjects. Sponsor approval and continuing review were obtained through a central IRB (New England Independent Review Board, NEIRB No. 120160610). For academic investigative sites that did not receive a waiver to use the central IRB, full board approval was obtained from the respective governing IRBs, and documentation of approval was submitted to CorEvitas, LLC, prior to initiating any study procedures.

### Study population

Eligible patients were adults (aged ≥ 18 years) who had a rheumatologist-confirmed diagnosis of RA and had initiated treatment with a TNFi (adalimumab, etanercept, certolizumab pegol, golimumab, or infliximab), an IL-6Ri (tocilizumab or sarilumab), or a JAKi (tofacitinib, baricitinib, or upadacitinib) during or after January 2010, until December 2019. In addition, patients were required to have both Hb and CRP data available at baseline and at month 6 (± 3 months) and to have had continued treatment with the same TNFi, IL-6Ri, or JAKi from baseline through to at least the month 6 (± 3 months) follow-up visit. Patients who discontinued therapy prior to follow-up were excluded.

### Assessments

Data were collected from rheumatologist and patient questionnaires completed during routine clinical encounters that occurred over the study period. These questionnaires were used to gather information on disease activity, comorbidities, use of medications including steroids, conventional synthetic DMARDs (csDMARDs), tsDMARDs, bDMARDs, and adverse events. As the CorEvitas RA registry is strictly an observational registry that reflects typical clinical practice, it does not mandate that laboratory tests, including CRP or Hb, are performed; however, it does solicit submission of laboratory results that were obtained as part of routine clinical care. Baseline patient and disease characteristics were recorded for each eligible patient.

### Outcomes

The mean changes in Hb and CRP concentration were evaluated for the three treatment groups, with adjusted changes from baseline to month 6 (± 3 months) being compared between the IL-6Ri treatment group and the TNFi and JAKi treatment groups. The proportions of patients whose Hb or CRP levels improved from abnormal (Hb: < 12 g/dL for women or < 13 g/dL for men [classified as anemia] [27]; CRP: ≥ 0.8 mg/dL) to normal levels and worsened from normal to abnormal levels relative to baseline levels were evaluated. In an additional analysis, changes in Hb levels were categorized as (i) increase (change ≥ 1.5 g/dL) or no significant change (defined as decrease < 1.5 g/dL or increase < 1.5 g/dL), (ii) mild decrease (decrease ≤ 1.5 g/dL), or (iii) moderate or worse decrease (> 1.5 g/dL); 1.5 g/dL has previously been used in the literature as a threshold for Hb change [28].

### Assessment of change in Hb and CRP levels

Unadjusted calculations of the mean changes in Hb and CRP levels from baseline to month 6 by drug class were undertaken. Adjusted mean changes from baseline to month 6, comparing IL-6Ri with TNFi and JAKi separately, were calculated using multiple linear regression, reporting the mean change (beta coefficient) and 95% confidence intervals (CI).

For Hb, the proportion of patients with low month 6 level, the proportion who changed from normal baseline to low month 6 level, and the proportion who changed from low baseline to normal month 6 level were reported by drug class; similarly, for CRP, the proportion of patients with high month 6 level, the proportion who changed from normal baseline to high month 6 level, and the proportion who changed from high baseline to normal month 6 level were reported by drug class. The odds of patients being in these Hb or CRP change categories at month 6, comparing IL-6Ri with TNFi and JAKi separately, were calculated by multiple logistic regression, reporting odds ratios (OR) and 95% CI.

In an additional analysis for Hb levels, the odds of having a mild or moderate/worse decrease in Hb level by month 6 versus having an increase or no change, comparing IL-6Ri with TNFi and JAKi separately, were calculated by multiple logistic regression, reporting OR and 95% CI. Finally, for CRP, the proportion of patients who had normal levels (≤ 0.3 mg/dL) at month 6 was reported, by drug class and for the strata of patients with CRP levels > 0.3 mg/dL at baseline.

Multiple regression models were adjusted for covariates that were imbalanced at baseline. For Hb analyses, adjusted model covariates were baseline Hb, age, duration of RA, morning stiffness duration, sex, current smoker status, prior use of one csDMARD, prior use of a non-TNFi bDMARD, white race, cyclic citrullinated peptide antibody positivity, combination treatment with methotrexate, and Clinical Disease Activity Index (CDAI; baseline CDAI and 6-month CDAI). For CRP analyses, adjusted model covariates were baseline CRP, age, duration of RA, EuroQol-5 Dimension score, Health Assessment Questionnaire (HAQ) score, sex, prior use of ≥ 2 TNFi, white race, history of hyperlipidemia, and CDAI (baseline CDAI and 6-month CDAI). Adjustments across abnormal and normal baseline Hb/CRP levels were not assessed.

## Results

### Baseline characteristics

**Fig. 1:**
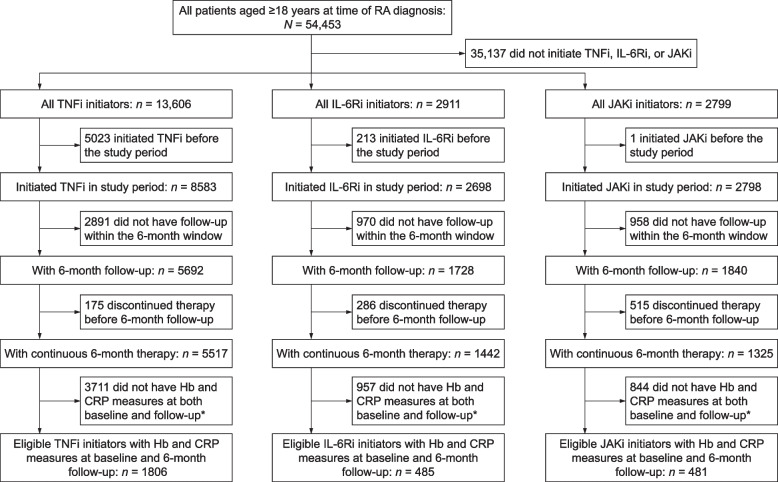
Selection of eligible patients from the CorEvitas RA registry who initiated treatment between January 2010 and December 2019. *Laboratory monitoring is not mandated in this observational registry; see the “Methods” section. CRP, C-reactive protein; Hb, hemoglobin; IL-6Ri, interleukin-6 receptor inhibitor; JAKi, Janus kinase inhibitor; RA, rheumatoid arthritis; TNFi, tumor necrosis factor inhibitor

Patients (*N* = 2772) who had initiated index treatment at baseline, had received 6-month continuous treatment, and had Hb and CRP measures at both baseline and follow-up were included in this analysis (TNFi, 65% [*n* = 1806]; IL-6Ri, 17% [*n* = 485]; JAKi, 17% [*n* = 481]; Fig. 1).

Many baseline demographic and disease characteristics differed between the treatment groups (Table 1). On average, patients in the TNFi group were younger (57.6 years) than those in the IL-6Ri or JAKi group (57.9 and 60.3 years, respectively; *p* < 0.001 among the groups). The mean duration of RA ranged from 8.6 years in the TNFi treatment group to 12.6 years in the IL-6Ri group and 13.3 years in the JAKi group (*p* < 0.001 among the groups). Patients in the IL-6Ri initiators group had the highest mean baseline scores for CDAI and HAQ. Patients in the TNFi group were most likely to be initiating the treatment as a first-line biologic, whereas the majority of patients in the IL-6Ri and JAKi initiators groups were third-or-higher-line biologic initiators.

**Table 1:** Baseline characteristics by treatment group

	TNFi (***N*** = 1806)^a^	IL-6Ri (***N*** = 485)^b^	JAKi (***N*** = 481)^c^	***p*** value for crude group comparison
Age, years, mean (SD)	57.6 (12.9)	57.9 (13.1)	60.3 (12.1)	< 0.001
Duration of RA, years, mean (SD)	8.6 (8.9)	12.6 (9.4)	13.3 (10.0)	< 0.001^d^
Female, *n* (%)	1405 (77.8)	393 (81.0)	391 (81.3)	0.12
White race, *n* (%)	1492 (82.9)	401 (83.4)	410 (86.5)	0.17
No insurance, *n* (%)	24 (1.3)	N/A	5 (1.0)	0.48^d^
Current smoker, *n* (%)	332 (18.5)	75 (15.6)	88 (18.4)	
BMI, mean (SD)	30.8 (7.6)	30.5 (7.4)	30.6 (7.1)	0.69
Seropositivity, *n*/*N* (%)^e^				
Anti-CCP antibody positive	480/1059 (45.3)	127/275 (46.2)	143/286 (50.0)	0.37
RF positive	779/1204 (64.7)	187/306 (61.1)	198/307 (64.5)	0.50
Hb, g/dL, mean (SD)	13.46 (1.41)	13.58 (1.37)	13.19 (1.48)	
Normal Hb, *n* (%)^f^	1505 (83.3)	392 (80.8)	391 (81.3)	
Abnormal Hb, *n* (%)^f^	301 (16.7)	93 (19.2)	90 (18.7)	
CRP, mg/dL, mean (SD)	0.89 (1.99)	0.63 (2.59)	1.01 (2.94)	
Normal CRP, *n* (%)^f^	1112 (61.6)	279 (57.5)	317 (65.9)	
Abnormal CRP, *n* (%)^f^	694 (38.4)	206 (42.5)	164 (34.1)	
History of comorbidities, *n* (%)				
CVD^g^	192 (10.6)	58 (12.0)	60 (12.5)	0.44
Malignancy^h^	111 (6.1)	37 (7.6)	37 (7.7)	0.31
Serious infections^i^	143 (7.9)	43 (8.9)	50 (10.4)	0.21
CDAI, mean (SD)	17.7 (12.9)	20.1 (12.9)	18.3 (12.9)	< 0.001
CDAI category, *n* (%)				
Remission (< 2.8)	169 (9.4)	20 (4.2)	40 (8.3)	0.003
Low (≥ 2.8 to < 10)	442 (24.6)	102 (21.2)	117 (24.3)	
Moderate (≥ 10 to < 22)	621 (34.5)	180 (37.4)	159 (33.1)	
High ( ≥ 22)	568 (31.6)	179 (37.2)	165 (34.3)	
HAQ, mean (SD)	0.9 (0.7)	1.1 (0.7)	1.0 (0.7)	< 0.001
No prednisone use, *n* (%)	1272 (70.4)	313 (64.5)	347 (72.1)	0.10
Concomitant therapy as monotherapy, *n* (%)^j^	346 (19.2)	159 (32.8)	177 (36.8)	< 0.001
Biologic line of therapy, *n* (%)				
First	1388 (76.9)	295 (60.8)	313 (65.1)	< 0.001^k^
Second	223 (12.3)	57 (11.8)	46 (9.6)	
Third or higher	195 (10.8)	133 (27.4)	122 (25.4)	

*BMI* body mass index, *CCP* cyclic citrullinated peptide, *CDAI *Clinical Disease Activity Index, *CRP* C-reactive protein, *CVD* cardiovascular disease, *HAQ* Health Assessment Questionnaire, *Hb* hemoglobin, *IL-6Ri* interleukin-6 receptor inhibitor, *JAKi* Janus kinase inhibitor, *N/A* not applicable, *RA* rheumatoid arthritis, *RF* rheumatoid factor, *SD* standard deviation, *TNFi* tumor necrosis factor inhibitor

^a^Certolizumab pegol, *n* = 269; etanercept, *n* = 416; adalimumab, *n* = 522; infliximab, *n* = 280; golimumab, *n* = 319

^b^Tocilizumab, *n* = 433; sarilumab, *n* = 52

^c^Baricitinib, *n* = 33; upadacitinib, *n* = 9; tofacitnib, *n* = 439

^d^Non-parametric test was used: the Fisher exact test for categorical variables and Kruskal–Wallis test for continuous variables (for all other characteristics in the table, chi-square tests were used for categorical variables and one-way analysis of variance for continuous variables)

^e^Laboratory monitoring is not mandated in this observational registry

^f^Normal defined as Hb ≥ 12 g/dL for females and Hb ≥ 13 g/dL for males and CRP < 0.8 mg/dL

^g^History of CVD includes myocardial infarction, stroke, acute coronary syndrome, coronary artery disease, coronary heart failure, revascularization procedure including percutaneous coronary intervention, coronary artery bypass grafting or coronary artery stents, ventricular arrhythmia, cardiac arrest, unstable angina, peripheral arterial disease, other cardiovascular events, pulmonary embolism, carotid artery disease, deep vein thrombosis, and transient ischemic attack

^h^History of malignancy includes lymphoma, lung cancer, breast cancer, non-melanoma skin cancer, and other cancers

^i^Serious infections include infections that led to hospitalization or intravenous antibiotics: joint/bursa, cellulitis, sinusitis, diverticulitis, sepsis, pneumonia, bronchitis, gastroenteritis, meningitis, urinary tract infection, upper respiratory tract infection, or infection of other specified sites

^j^Concomitant therapy excludes any disease-modifying anti-rheumatic drug

^k^In addition, there was a statistically significant difference (*p* < 0.001) for the IL-6Ri and JAKi groups combined, compared with the TNFi group

### Hemoglobin and C-reactive protein

Of the 2772 patients included in the analysis, 1476 (53%) had normal Hb and CRP values at initiation, 1044 (38%) had either abnormal Hb or CRP, and 252 (9%) had abnormal Hb and CRP at initiation (see Additional file 1). Baseline characteristics by Hb status or CRP status, further stratified by drug class, are also presented in Additional file 2.

#### Outcomes at month 6

##### Hemoglobin

**Fig. 2:**
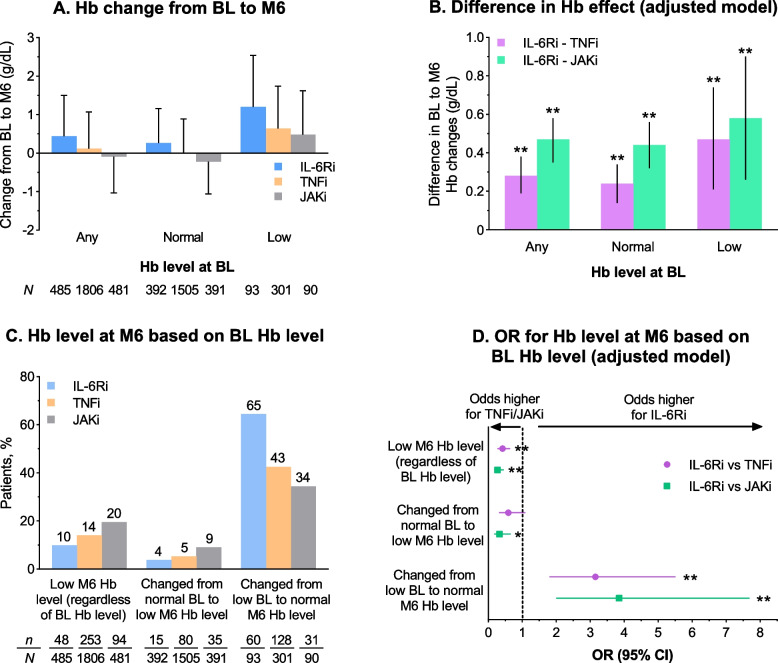
Assessment of change in Hb level: crude mean (± SD) change in Hb level from baseline to month 6 (**A**). Adjusted mean change (beta coefficient ± 95% CI) in Hb level from baseline to month 6, comparing IL-6Ri with TNFi and JAKi separately (**B**). Proportions of patients with low month 6 Hb level, proportions who changed from normal baseline to low month 6 Hb level, and proportions who changed from low baseline to normal month 6 Hb level (**C**). Odds of having a low month 6 Hb level, changing from normal baseline to low month 6 Hb level, or changing from low baseline to normal month 6 Hb level, comparing IL-6Ri with TNFi and JAKi separately (**D**). *p < 0.01; **p < 0.001. Low Hb was defined as < 12 g/dL (women) or < 13 g/dL (men). Adjusted model covariates were baseline Hb, age, duration of rheumatoid arthritis, morning stiffness duration, sex, current smoker status, prior use of one conventional synthetic disease-modifying anti-rheumatic drug, prior use of a non-TNFi biologic disease-modifying anti-rheumatic drug, white race, cyclic citrullinated peptide antibody positivity, combination therapy with methotrexate, and CDAI (baseline CDAI and 6-month CDAI). BL, baseline; CDAI, Clinical Disease Activity Index; CI, confidence interval; Hb, hemoglobin; IL-6Ri, interleukin-6 receptor inhibitor; JAKi, Janus kinase inhibitor; M, month; OR, odds ratio; SD, standard deviation; TNFi, tumor necrosis factor inhibitor

The overall change in Hb level from baseline to month 6 (± 3 months) (i.e., the 6-month value minus baseline value) observed in the IL-6Ri treatment group was 0.44 ± 1.06 g/dL compared with 0.12 ± 0.95 g/dL in the TNFi group and − 0.09 ± 0.94 g/dL in the JAKi group (Fig. 2A). A similar trend for a greater increase in Hb levels was observed for IL-6Ri compared with TNFi or JAKi when changes from baseline to month 6 were assessed separately for low or normal baseline Hb levels. Comparison of adjusted mean differences from baseline to month 6 between the treatment groups showed statistically significantly greater improvements in Hb levels in the IL-6Ri group compared with other treatment groups, regardless of baseline Hb levels (*p* < 0.001; Fig. 2B). In addition, the proportion of patients who had a low Hb level at baseline and achieved a normal Hb level at month 6 was higher in the IL-6Ri group (65%) than the TNFi (43%) and JAKi (34%) groups (Fig. 2C). The overall proportion of patients whose Hb level was low after 6 months of treatment was lower for IL-6Ri (10%) than for TNFi (14%) and JAKi (20%). Patients in the IL-6Ri group had significantly higher odds of attaining normal Hb levels at month 6 from low Hb levels at baseline compared with those in the TNFi and JAKi groups (adjusted ORs 3.15 and 3.85, respectively; both *p* < 0.001; Fig. 2D).

**Fig. 3:**
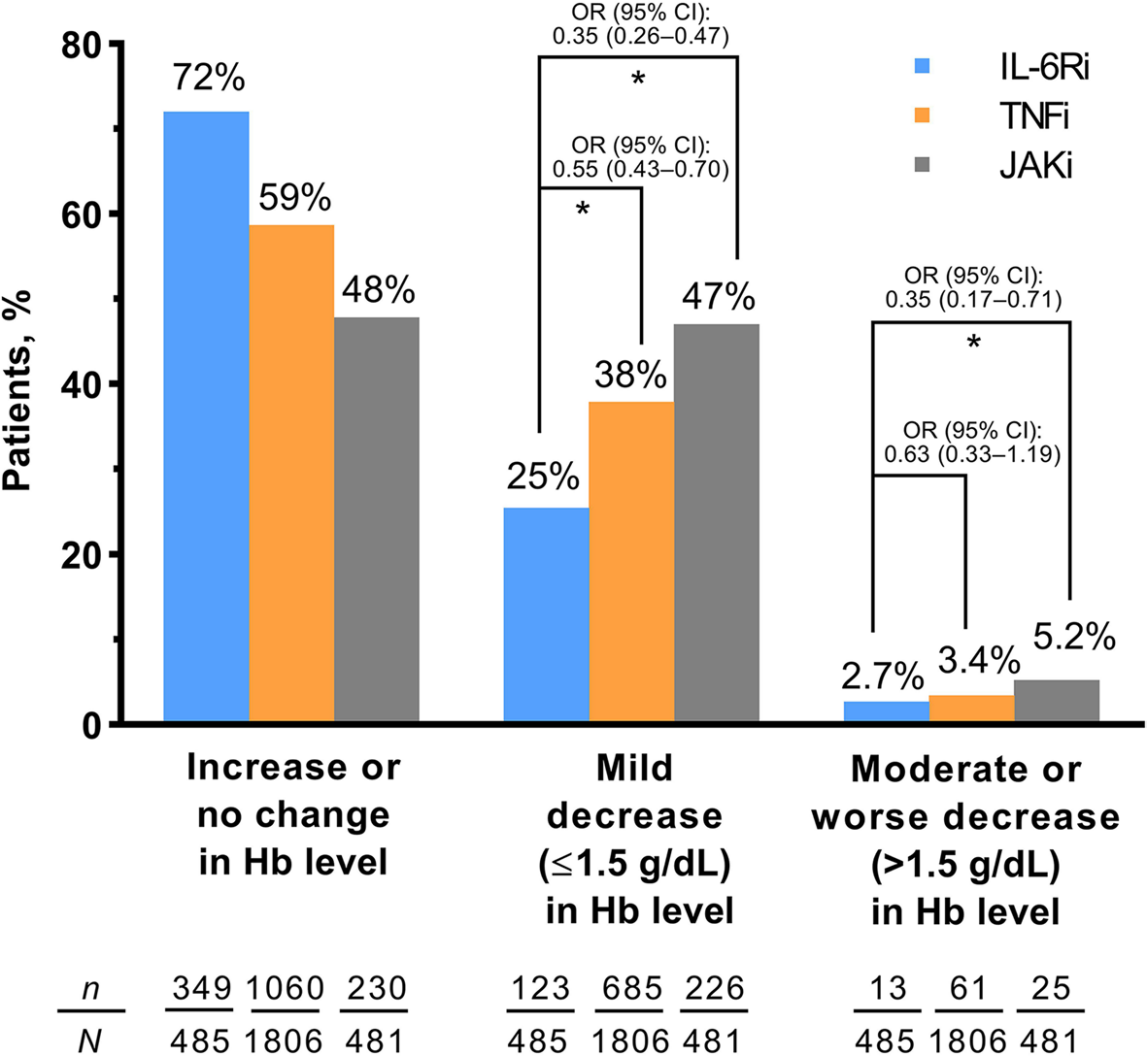
Proportion of patients with an increase or no change, mild decrease, or moderate/worse decrease in Hb level at month 6, with adjusted OR and 95% CI comparing IL-6Ri with TNFi and JAKi separately. *p < 0.001. The OR reported are from adjusted analyses. Adjusted model covariates were baseline Hb, age, duration of rheumatoid arthritis, morning stiffness duration, sex, current smoker status, prior use of one conventional synthetic disease-modifying anti-rheumatic drug, prior use of a non-TNFi biologic disease-modifying anti-rheumatic drug, white race, cyclic citrullinated peptide antibody positivity, and combination therapy with methotrexate. CI, confidence interval; Hb, hemoglobin; IL-6Ri, interleukin-6 receptor inhibitor; JAKi, Janus kinase inhibitor; OR, odds ratio; TNFi, tumor necrosis factor inhibitor

Patients in the IL-6Ri group had statistically significantly lower odds of experiencing a mild decrease in Hb levels (≤ 1.5 g/dL; OR 0.55 vs TNFi and 0.35 vs JAKi; *p* < 0.001 for both), non-significantly lower odds of experiencing a moderate or worse Hb decrease versus TNFi (> 1.5 g/dL; OR 0.63, *p* = 0.16), and significantly lower odds of moderate or worse Hb decrease versus JAKi (OR 0.35; *p* < 0.001), compared with no change or an increase in Hb (Fig. 3). Consistent findings were observed when the same analysis was conducted for the subgroup of patients who had moderate or high CDAI at month 6 and for the subgroup with low CDAI or remission at month 6 (Additional file 3).

There were only two patients with Hb ≤ 8 g/dL at baseline, one each initiating a TNFi and an IL-6Ri. At the 6-month follow-up, both had low Hb, with one (the IL-6Ri initiator) experiencing an improvement in CRP to a normal level.

##### C-reactive protein

**Fig. 4:**
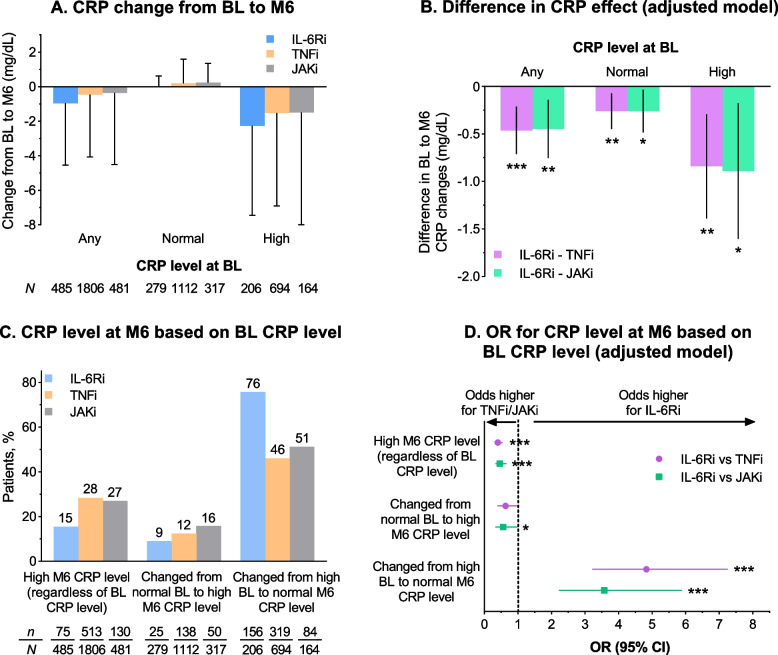
Assessment of change in CRP level: crude mean (± SD) change in CRP level from baseline to month 6 (**A**). Adjusted mean change (beta coefficient ± 95% CI) in CRP level from baseline to month 6, comparing IL-6Ri with TNFi and JAKi separately (**B**). Proportions of patients with high month 6 CRP level, proportions who changed from normal baseline to high month 6 CRP level, and proportions who changed from high baseline to normal month 6 CRP level (**C**). Odds of having a high month 6 CRP level, changing from normal baseline to high month 6 CRP level, or changing from high baseline to normal month 6 CRP level, comparing IL-6Ri with TNFi and JAKi separately (**D**). *p < 0.05; **p < 0.01; ***p < 0.001. High CRP was defined as ≥ 0.8 mg/dL. Adjusted model covariates were baseline CRP, age, duration of rheumatoid arthritis, EuroQol-5 Dimension score, Health Assessment Questionnaire score, sex, prior use of ≥ 2 TNFi, white race, history of hyperlipidemia, and CDAI (baseline CDAI and 6-month CDAI). BL, baseline; CDAI, Clinical Disease Activity Index; CI, confidence interval; CRP, C-reactive protein; IL-6Ri, interleukin-6 receptor inhibitor; JAKi, Janus kinase inhibitor; M, month; OR, odds ratio; SD, standard deviation; TNFi, tumor necrosis factor inhibitor

The overall decrease in CRP level from baseline to month 6 (± 3 months) observed in the IL-6Ri group was greater than in the TNFi and JAKi groups (Fig. 4A). Comparison of adjusted mean differences from baseline to month 6 showed statistically significantly greater reductions in CRP levels with IL-6Ri for any baseline CRP level (*p* < 0.001 vs TNFi and *p* < 0.01 vs JAKi; Fig. 4B). Statistically significantly greater reductions were reported for the IL-6Ri group versus the TNFi and JAKi groups at normal and high baseline CRP levels (*p* < 0.05). In addition, the proportion of patients who achieved a change in CRP level from high to normal at month 6 was higher in the IL-6Ri group (76%) than in the TNFi (46%) and JAKi (51%) groups (Fig. 4C). The overall proportion of patients with a high CRP level at month 6 was lower in the IL-6Ri group (15%) than in the TNFi (28%) and JAKi (27%) groups (Fig. 4C). Patients in the IL-6Ri group had statistically significantly higher odds of attaining normal CRP levels at month 6 from high baseline CRP levels compared with those in the TNFi and JAKi groups (*p* < 0.001; Fig. 4D).

**Fig. 5:**
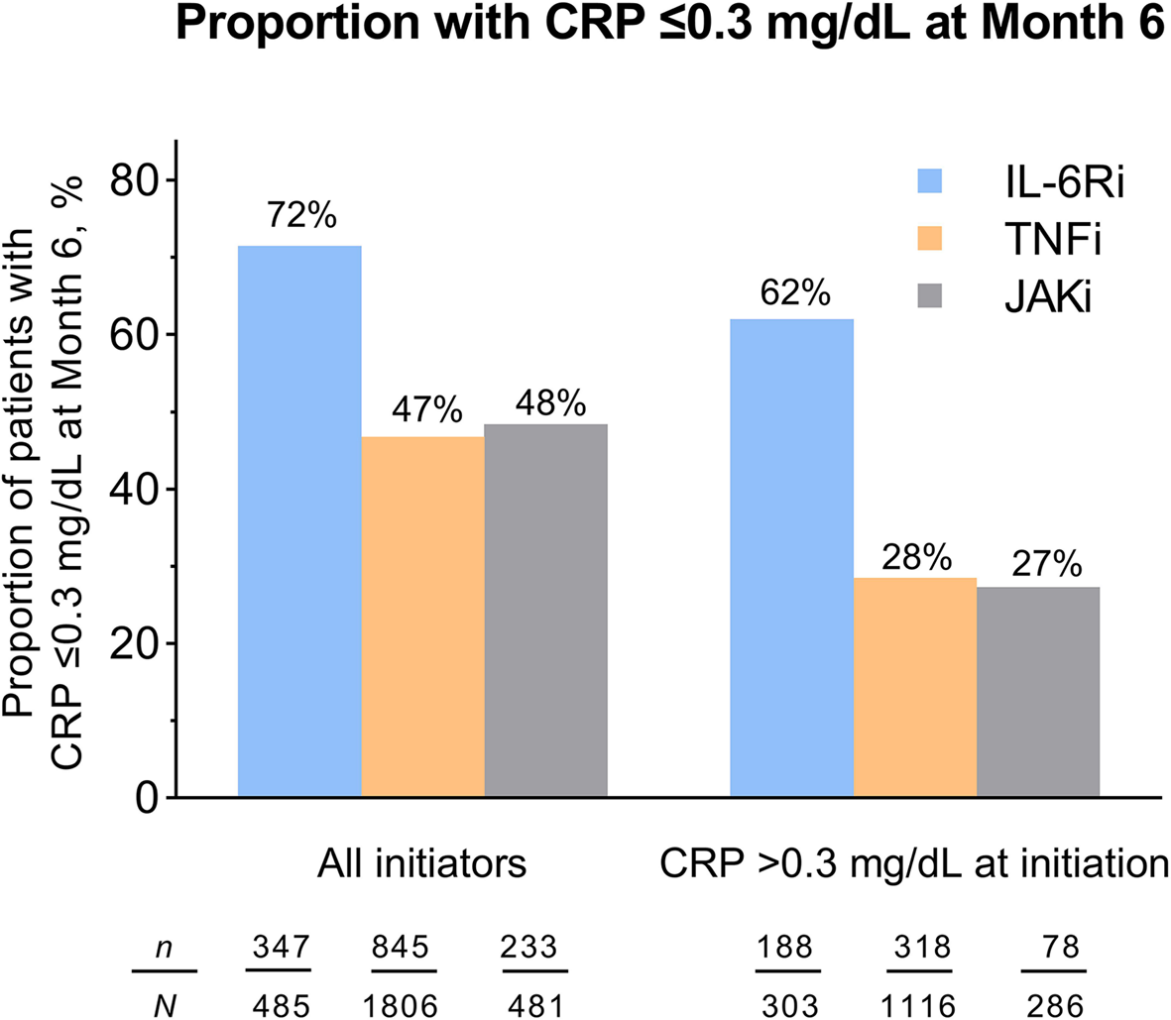
Proportion of patients with CRP level ≤ 0.3 mg/dL at month 6. CRP, C-reactive protein; IL-6Ri, interleukin-6 receptor inhibitor; JAKi, Janus kinase inhibitor; TNFi, tumor necrosis factor inhibitor

The proportion of patients whose CRP level was ≤ 0.3 mg/dL at month 6 was higher in the IL-6Ri group (72%) than in the TNFi (47%) and JAKi (48%) groups. Among patients whose CRP at treatment initiation was > 0.3 mg/dL, those proportions were 62%, 28%, and 27% (Fig. 5).

## Discussion

In this analysis of real-world data from a large US RA registry, we observed that 6 months of continuous therapy with an IL-6R inhibitor was associated with improvements in Hb and CRP levels, regardless of the level at baseline, which were greater than improvements achieved with continuous 6-month therapy with either a TNF inhibitor or a JAK inhibitor. In addition, a greater proportion of patients who had abnormal Hb or CRP levels at baseline achieved normal levels at month 6 after therapy with IL-6 inhibition compared with TNF inhibitor or JAK inhibitor users.

While the number of studies evaluating the effects of these agents on Hb and CRP levels is limited, our results agree with previous findings indicating that IL-6R inhibitors have the greatest benefit in improving Hb levels in patients with RA. A post hoc analysis of data from the phase 3 MONARCH study found that treatment with sarilumab resulted in significantly greater increases in Hb than treatment with the TNF inhibitor adalimumab [20]. In a smaller trial, treatment with the IL-6R inhibitor tocilizumab was associated with greater reductions in serum hepcidin-25 than treatment with the TNF inhibitor infliximab. The reduction in serum hepcidin observed in the infliximab group was accompanied by a decrease in serum IL-6, allowing for the possibility that the effect of TNF-α inhibition on the reduction of hepcidin-25 levels was indirect, occurring via IL-6 inhibition [15]. Another study of patients from the Japanese KURAMA database demonstrated a significantly greater increase of Hb in the tocilizumab group than in the non-tocilizumab group, with a significant association between the increase in Hb levels and lower Hb and higher CRP levels at baseline, as well as with a greater reduction in disease activity (CDAI) [23].

As noted, the greater improvements in Hb and CRP levels with IL-6R inhibitor treatment than with TNF inhibitor or JAK inhibitor treatment observed in our analysis are consistent with the IL-6 mechanism of action: an increase in serum IL-6 results in increased production of hepcidin (which reduces the levels of available iron and therefore Hb) and CRP in hepatocytes [29]. Furthermore, the observed greater improvement in Hb with IL-6R versus JAK inhibition might be due to a reduction of erythropoiesis via JAK2 inhibition, causing some of the beneficial effects of reducing inflammation (i.e., increased Hb) to be partially offset [30]. Although the IL-6 pathway can be inhibited by either IL-6R or JAK inhibitors [31], our data suggest a more pronounced effect on anemia and inflammation with an anti-IL-6R treatment.

The findings of this study have potential clinical implications. In a recent analysis of patients from a phase 3 trial, a higher risk of joint damage progression over 1 year, as assessed by radiographic imaging, was reported for patients with low baseline Hb; in both patients with low and normal baseline Hb from that study, sarilumab improved the radiological outcomes compared with placebo over 1 year [32]. Furthermore, some studies suggest an association between improvements in Hb and patient-reported outcomes, including energy levels and quality of life [11]. CRP is a key marker of inflammation, with increased levels being associated with a higher incidence of CVD [33, 34]. In patients with RA, elevated CRP levels have been associated with increased risk for many comorbidities, including CVD [19]. In the general population, an increased risk of CVD with CRP > 0.3 mg/dL has been observed [35]; a similar increase in CVD risk has also been observed in patients with RA [36]. In addition, an analysis of data from a phase 3 study of sarilumab and adalimumab showed that patients receiving either of these drugs over 24 weeks who achieve CRP ≤ 0.3 mg/dL at week 12 have improved quality of life, as assessed by patient-reported outcomes in the areas of pain, morning stiffness, fatigue, and sleep, at week 24 [37].

This study has several limitations. First, due to the observational nature of the registry, the assignment of patient treatment is made by their physician; therefore, patients may receive treatment based on the differences in underlying prognostic factors (channeling). While the analyses were adjusted for known differences across treatment groups, unmeasured confounding may still be present in the data. We did not include a variance adjustment for the possibility that patients may contribute multiple observations to the analysis. In addition, the three drug classes varied in the proportions who remained on the therapy to 6-month follow-up, out of all patients who had initiated that therapy in the study period (TNFi, 64%; IL-6Ri, 53%; JAKi, 47%). Since our study included only patients who remained on therapy, we might have selected patients who had more favorable outcomes on therapies, and we could have excluded patients who either did not respond to therapy or had an adverse event, including an abnormality in laboratory values. This was a completer’s analysis, focusing only on those who remained on therapy through the 6-month follow-up visit and who had laboratory values at both time points. As noted, the CorEvitas observational RA registry does not mandate specific laboratory analyses. Laboratory monitoring decisions are left to the discretion of the investigator. It is therefore possible that the selection of patients for measurement of these laboratory parameters could represent ascertainment or information bias, that is, patients who have laboratory abnormalities are more likely to have laboratory testing or to have reported abnormalities in the registry.

## Conclusions

To the best of our knowledge, this investigation is the first real-world analysis comparing the effects of multiple classes of targeted DMARDs on systemic inflammation and Hb levels, with results that align with the proposed mechanism of IL-6R inhibition. Our findings may be useful when considering treatment options for patients with RA.

## Supplementary Information

**Additional file 1.** Baseline characteristics by hemoglobin and CRP status at treatment initiation.

**Additional file 2.** (i) Baseline characteristics by hemoglobin status at treatment initiation, stratified by treatment group and (ii) baseline characteristics by CRP status at treatment initiation, stratified by treatment group.

**Additional file 3.** Proportion of patients with increase or no change, mild decrease, or moderate/worse decrease in Hb level at Month 6, with adjusted OR and 95% CI comparing IL-6Ri with TNFi and JAKi separately: (i) patients who had moderate or high CDAI at month 6 and (ii) patients who had low CDAI or remission at month 6.

**Additional file 4.** The RECORD statement.

## Data Availability

Data are available from CorEvitas, LLC through a commercial subscription agreement and are not publicly available. No additional data are available from the authors.

## Abbreviations

bDMARD: Biologic disease-modifying anti-rheumatic drug
BMI: Body mass index
CCP: Cyclic citrullinated peptide
CDAI: Clinical Disease Activity Index
CI: Confidence interval
CRP: C-reactive protein
csDMARD: Conventional synthetic disease-modifying anti-rheumatic drug
CVD: Cardiovascular disease
HAQ: Health Assessment Questionnaire
Hb: Hemoglobin
IL: Interleukin
IL-6Ri: Interleukin-6 receptor inhibitor(s)
IRB: Institutional review board
JAKi: Janus kinase inhibitor(s)
OR: Odds ratio
RA: Rheumatoid arthritis
RF: Rheumatoid factor
SD: Standard deviation
TNFi: Tumor necrosis factor inhibitor(s)
TNF-α: Tumor necrosis factor alpha
tsDMARDs: Targeted synthetic disease-modifying anti-rheumatic drug
