# Supplementary material for: The effect of targeted rheumatoid arthritis therapeutics on systemic inflammation and anemia: analysis of data from the CorEvitas RA registry
Source: Arthritis Res Ther. 2022 Dec 21;24:276. doi: 10.1186/s13075-022-02955-y (PMC9769058; doi:10.1186/s13075-022-02955-y)
Supplement: Supplementary file 1 — Additional file 1. Baseline characteristics by hemoglobin and CRP status at treatment initiation. [file 13075_2022_2955_MOESM1_ESM.docx]

**Additional file 1**

**Baseline characteristics by hemoglobin and CRP status at treatment initiation.**

|  | **Normal Hb and CRP**  ***N*=1476** | **Abnormal Hb or CRP**  ***N*=1044** | **Abnormal Hb and CRP**  ***N*=252** | ***p* value for crude  group comparison** |
| --- | --- | --- | --- | --- |
| Age, years, mean (SD) | 57.8 (12.4) | 58.0 (13.1) | 60.6 (14.0) | 0.001^a^ |
| Duration of RA, years, mean (SD) | 10.2 (9.4) | 9.8 (9.3) | 10.8 (9.7) | 0.33 |
| Female, *n* (%) | 1167 (79.1) | 829 (79.4) | 193 (76.6) | 0.78 |
| White race, *n* (%) | 1249 (85.1) | 861 (83.2) | 193 (76.9) | 0.11 |
| No insurance, *n* (%) | 13 (0.9) | 15 (1.4) | <5 | 0.96 |
| Current smoker, *n* (%) | 253 (17.2) | 200 (19.3) | 42 (16.8) |  |
| BMI, mean (SD) | 29.7 (6.5) | 32.1 (8.2) | 31.7 (8.5) | <0.001^a^ |
| Seropositivity: CCP+ and/or RF+, *n* (%)^b^ | 692 (46.9) | 534 (51.1) | 140 (55.6) | 0.01 |
| History of comorbidities, *n* (%) |  |  |  |  |
| CVD^c^ | 154 (10.4) | 113 (10.8) | 43 (17.1) | 0.23 |
| Malignancy^d^ | 112 (7.6) | 57 (5.5) | 16 (6.3) | 0.66 |
| Serious infections^e^ | 105 (7.1) | 104 (10.0) | 27 (10.7) | 0.39 |
| CDAI, mean (SD) | 16.5 (12.2) | 19.4 (13.1) | 23.0 (14.5) | <0.001^a^ |
| CDAI category, *n* (%) |  |  |  |  |
| Remission (<2.8) | 156 (10.6) | 62 (6.0) | 11 (4.4) | <0.001 |
| Low (≥2.8 to <10) | 394 (26.8) | 226 (21.7) | 41 (16.4) |  |
| Moderate (≥10 to <22) | 500 (34.0) | 381 (36.6) | 79 (31.6) |  |
| High (≥22) | 420 (28.6) | 373 (35.8) | 119 (47.6) |  |
| HAQ, mean (SD) | 0.8 (0.7) | 1.0 (0.7) | 1.3 (0.7) | <0.001 |
| No prednisone use, *n* (%) | 1073 (72.7) | 706 (67.6) | 153 (60.7) | 0.002 |
| Concomitant therapy: as monotherapy, *n* (%)^f^ | 384 (26.0) | 246 (23.6) | 52 (20.6) | 0.04 |
| Biologic line of therapy, *n* (%) |  |  |  |  |
| First | 1050 (71.1) | 757 (72.5) | 196 (77.8) | 0.24 |
| Second | 186 (12.6) | 118 (11.3) | 22 (8.7) |  |
| Third or higher | 240 (16.3) | 169 (16.2) | 34 (13.5) |  |

^a^Non-parametric test was used: Fisher exact test for categorical variables and Kruskal–Wallis test for continuous variables (for all other characteristics in the table, chi-square tests were used for categorical variables and one-way analysis of variance for continuous variables).

^b^Laboratory monitoring is not mandated in this observational registry.

^c^History of CVD includes myocardial infarction, stroke, acute coronary syndrome, coronary artery disease, coronary heart failure, revascularization procedure including percutaneous coronary intervention, coronary artery bypass grafting or coronary artery stents, ventricular arrhythmia, cardiac arrest, unstable angina, peripheral arterial disease, other cardiovascular event, pulmonary embolism, carotid artery disease, deep vein thrombosis, and transient ischemic attack.

^d^History of malignancy includes lymphoma, lung cancer, breast cancer, non-melanoma skin cancer, and other cancer.

^e^Serious infections include infections that led to hospitalization or intravenous antibiotics: joint/bursa, cellulitis, sinusitis, diverticulitis, sepsis, pneumonia, bronchitis, gastroenteritis, meningitis, urinary tract infection, upper respiratory tract infection, or infection of other specified sites.

^f^Concomitant therapy excludes any disease-modifying anti-rheumatic drug.

BMI, body mass index; CDAI, Clinical Disease Activity Index; CCP, cyclic citrullinated peptide; CRP, C-reactive protein; CVD, cardiovascular disease; HAQ, Health Assessment Questionnaire; Hb, hemoglobin; RA, rheumatoid arthritis; RF, rheumatoid factor; SD, standard deviation.
