# Supplementary material for: The effect of targeted rheumatoid arthritis therapeutics on systemic inflammation and anemia: analysis of data from the CorEvitas RA registry
Source: Arthritis Res Ther. 2022 Dec 21;24:276. doi: 10.1186/s13075-022-02955-y (PMC9769058; doi:10.1186/s13075-022-02955-y)
Supplement: Supplementary file 3 — Additional file 3. Proportion of patients with increase or no change, mild decrease, or moderate/worse decrease in Hb level at Month 6, with adjusted OR and 95% CI comparing IL-6Ri with TNFi and JAKi separately: (i) patients who had moderate or high CDAI at month 6 and (ii) patients who had low CDAI or remission at month 6. [file 13075_2022_2955_MOESM3_ESM.docx]

**Additional file 3**

**Proportion of patients with increase or no change, mild decrease, or moderate/worse decrease in Hb level at Month 6, with adjusted OR and 95% CI comparing IL-6Ri with TNFi and JAKi separately: patients who had moderate or high CDAI at month 6**

*p<0.01; **p<0.001.

Moderate or high CDAI was defined as CDAI >10. Odds ratios reported are from adjusted analyses. Adjusted model covariates were baseline Hb, age, duration of rheumatoid arthritis, morning stiffness duration, baseline CDAI, 6-month CDAI, sex, current smoker status, prior use of one conventional synthetic disease-modifying anti-rheumatic drug, prior use of a non-TNFi biological disease-modifying anti-rheumatic drug, white race, cyclic citrullinated peptide antibody positivity, and combination therapy with methotrexate.

CI, confidence interval; Hb, hemoglobin; IL-6Ri, interleukin-6 receptor inhibitor; JAKi, Janus kinase inhibitor; OR, odds ratio; TNFi, tumor necrosis factor inhibitor.

**Proportion of patients with increase or no change, mild decrease, or moderate/worse decrease in Hb level at Month 6, with adjusted OR and 95% CI comparing IL-6Ri with TNFi and JAKi separately: patients who had low CDAI or remission at month 6**

**p<0.001.

Low CDAI or remission was defined as CDAI ≤10. Odds ratios reported are from adjusted analyses. Adjusted model covariates were baseline Hb, age, duration of rheumatoid arthritis, morning stiffness duration, baseline CDAI, 6-month CDAI, sex, current smoker status, prior use of one conventional synthetic disease-modifying anti-rheumatic drug, prior use of a non-TNFi biological disease-modifying anti-rheumatic drug, white race, cyclic citrullinated peptide antibody positivity, and combination therapy with methotrexate.

CI, confidence interval; Hb, hemoglobin; IL-6Ri, interleukin-6 receptor inhibitor; JAKi, Janus kinase inhibitor; OR, odds ratio; TNFi, tumor necrosis factor inhibitor.
